# High-Frequency Repetitive Transcranial Magnetic Stimulation (rTMS) Improves Functional Recovery by Enhancing Neurogenesis and Activating BDNF/TrkB Signaling in Ischemic Rats

**DOI:** 10.3390/ijms18020455

**Published:** 2017-02-20

**Authors:** Jing Luo, Haiqing Zheng, Liying Zhang, Qingjie Zhang, Lili Li, Zhong Pei, Xiquan Hu

**Affiliations:** 1 Department of Rehabilitation Medicine, The Third Affiliated Hospital, Sun Yat-Sen University, Guangzhou 510630, China; jill_272@foxmail.com (J.L.); zhenghaiqing0909@aliyun.com (H.Z.); zhangliying_good@126.com (L.Z.); zhangqingjie3415@163.com (Q.Z.); lovelyxt2008@163.com (L.L.); 2 Department of Neurology, The First Affiliated Hospital, Sun Yat-Sen University, Guangzhou 510080, China; peizhong@mail.sysu.edu.cn

**Keywords:** rTMS, neurological function, neural stem cells, BDNF, TrkB, MCAO

## Abstract

Repetitive transcranial magnetic stimulation (rTMS) has rapidly become an attractive therapeutic approach for stroke. However, the mechanisms underlying this remain elusive. This study aimed to investigate whether high-frequency rTMS improves functional recovery mediated by enhanced neurogenesis and activation of brain-derived neurotrophic factor (BDNF)/tropomyosin-related kinase B (TrkB) pathway and to compare the effect of conventional 20 Hz rTMS and intermittent theta burst stimulation (iTBS) on ischemic rats. Rats after rTMS were sacrificed seven and 14 days after middle cerebral artery occlusion (MCAO), following evaluation of neurological function. Neurogenesis was measured using specific markers: Ki67, Nestin, doublecortin (DCX), NeuN and glial fibrillary acidic protein (GFAP), and the expression levels of BDNF were visualized by Western blotting and RT-PCR analysis. Both high-frequency rTMS methods significantly improved neurological function and reduced infarct volume. Moreover, 20 Hz rTMS and iTBS significantly promoted neurogenesis, shown by an increase of Ki67/DCX, Ki67/Nestin, and Ki67/NeuN-positive cells in the peri-infarct striatum. These beneficial effects were accompanied by elevated protein levels of BDNF and phosphorylated-TrkB. In conclusion, high-frequency rTMS improves functional recovery possibly by enhancing neurogenesis and activating BDNF/TrkB signaling pathway and conventional 20 Hz rTMS is better than iTBS at enhancing neurogenesis in ischemic rats.

## 1. Introduction

Stroke is a major cause of neurological disability that leads to serious clinical consequences. It has been estimated that 90% of stroke survivors suffer permanent neurological deficits [1]. At present, rehabilitation therapy is the best approach for treating neurological deficits after stroke [2]. However, it is not ideal because most stroke survivors remain neurologically impaired after rehabilitation. Thus, there is an urgent need for the development of novel approaches to stroke rehabilitation. One promising strategy for stroke rehabilitation is to enhance endogenous pathways that support restoration after brain damage [3]. Neural stem cell (NSC) plays a key role in endogenous restoration following stroke [4]. NSC persists in the rostral subventricular zone (SVZ) and subgranular zone (SGZ) of the hippocampal dentate gyrus (DG) throughout life in mammals [5]. More importantly, NSC can proliferate and migrate into damaged brain regions following ischemic stroke [3,4,6]. Therefore, the promotion of endogenous neurogenesis is an attractive strategy for stroke rehabilitation.

Repetitive transcranial magnetic stimulation (rTMS) is a noninvasive neuromodulatory technique which affects brain physiology through magnetic pulses. Different rTMS techniques have been applied to stroke rehabilitation [7], and, interestingly, they can produce different modulatory effects. For example, high-frequency rTMS (>5 Hz) stimulates cortical excitability and generates long-term potentiation (LTP)-like effects. In contrast, low-frequency rTMS (<1 Hz) reduces cortical excitability and produces long-term depression (LTD) [8]. Intermittent theta burst stimulation (iTBS) is a novel form of high-frequency rTMS [9]. Conventional high-frequency rTMS procedures last between 20 and 45 min, as compared to TBS paradigms that require 1 to 3 min of stimulation [10]. Recently, some studies have suggested that iTBS applied to the ipsilateral side of human or animals has similar or better efficacy in treating stroke compared to conventional rTMS [11,12]. Whether iTBS is better than conventional high-frequency rTMS on improvement of functional recovery in ischemic rats is still unknown. Moreover, it is reported that high-frequency rTMS enhances neurogenesis in ischemic rats [13]. However, the underlying mechanism remains elusive.

One possible mechanism might be an increase in the expression of brain-derived neurotrophic factor (BDNF) after high-frequency rTMS [14]. BDNF is a member of the neurotrophin family, which has recently been shown to play a role both in protection and in recovery of function after stroke [15]. BDNF promotes NSC migration and proliferation via its receptor, tropomyosin-related kinase B (TrkB) [16,17]. Therefore, this study aimed to investigate whether the beneficial effect of rTMS on functional recovery is mediated via enhanced neurogenesis and activation of the BDNF-TrkB signaling pathway and to compare the effect of conventional 20 Hz rTMS and iTBS on neurogenesis in ischemic rats.

## 2. Results

### 2.1. rTMS Improves the Recovery of Neurological Function

**Figure 1:**
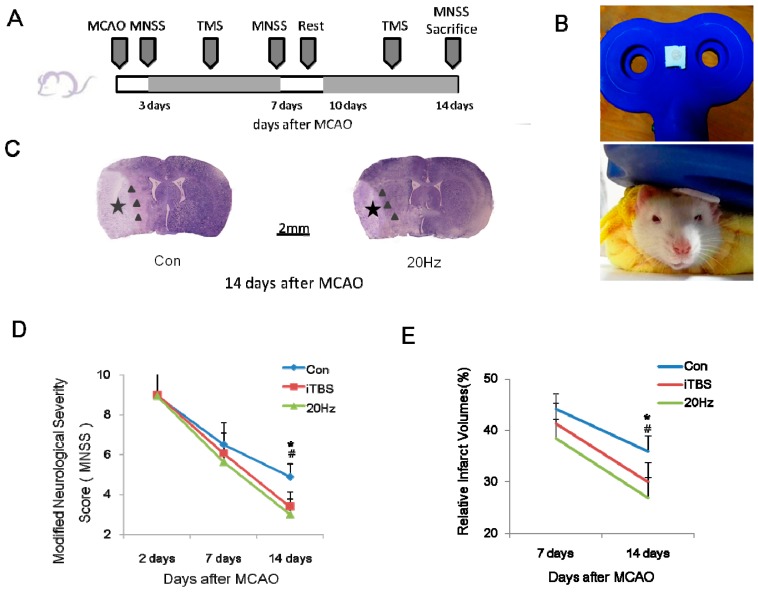
High-frequency repetitive transcranial magnetic stimulation (rTMS) following ischemic injury reduced the severity and volume of infarcts assessed 14 days later. (**A**) Experimental schedule; (**B**) Stimulation coil and method; (**C**) Representative examples of Nissl staining of brain tissues at 14 days after transient middle cerebral artery occlusion (MCAO). The location of the infarct area was indicated by a black star, and the peri-infarct striatum was indicated with black triangles; (**D**,**E**) Modified Neurological Severity Score (MNSS) values and infarct area volumes were reduced at 14 days, but not seven days, after MCAO in the intermittent theta burst stimulation (iTBS) and 20 Hz groups compared to controls. Values are mean ± SD; * *p* < 0.05 versus 20 Hz group; # *p* < 0.05 versus iTBS group. The scale bar in (**C**) applies to both slices shown in (**C**).

To investigate whether high-frequency rTMS improves functional recovery, we used the Modified Neurological Severity Score (MNSS) scale to assess neurological function in ischemic animals. MNSS values were zero in the sham-operated and sham + TMS group at any time point. A repeated measures analysis of variance (ANOVA) was used to assess the neurological function among the other three groups. It revealed a significant main effect of group (F2, 46 = 3.48, *p* < 0.05) and an effect of time (F2, 42 = 278.87, *p* < 0.05) at 14 days, but no significant interaction between the two factors (F4, 42 = 2.32, *p* > 0.05). In the control group, MNSS values were 8.94 ± 1.34, 6.5 ± 1.10, and 4.88 ± 0.64 at two, seven, and 14 days after middle cerebral artery occlusion (MCAO), respectively, indicating a spontaneous recovery after MCAO. In the 20 Hz group, the MNSS values were 8.94 ± 1.39, 5.63 ± 0.89, and 3.00 ± 0.76 at two, seven, and 14 days after MCAO, respectively. In the iTBS group, the MNSS were 9.00 ± 1.32, 6.06 ± 1.00, and 3.38 ± 0.74 at two, seven, and 14 days after MCAO, respectively. Rats receiving high-frequency rTMS (both 20 Hz and iTBS groups) exhibited significantly better recovery, as measured by lower MNSS values, than the control group at 14 days after MCAO (both *p* < 0.05). However, there were no significant differences in MNSS values among these three groups at two or seven days after MCAO (Figure 1D; all *p* > 0.05).

### 2.2. rTMS Reduces the Volume of the Infarct Area after an Ischemic Stroke

The infarct areas were zero in the sham-operated and sham + TMS group at any time point. At seven days after MCAO, relative infarct volumes did not differ between the control, iTBS, and 20 Hz groups (infarct volumes were 44.19% ± 2.99%, 41.29% ± 3.97%, and 38.46% ± 3.71%, respectively; all *p* > 0.05). However, after 14 days, the relative infarct volume was significantly reduced in the iTBS and 20 Hz groups compared to controls (30.02% ± 3.78%, 26.84% ± 4.02%, and 35.97% ± 3.02%, respectively; Figure 1E; both *p* < 0.05). Improvements in the MNSS were positively correlated with reductions in infarct volume (*r* = 0.957, *p* < 0.001). These findings suggested that high-frequency rTMS mitigated brain damage and improved neurological function in ischemic rats.

### 2.3. rTMS Increases the Migration of Neural Progenitor Cells in the Ipsilateral SVZ and Peri-Infarct Striatum

**Figure 2:**
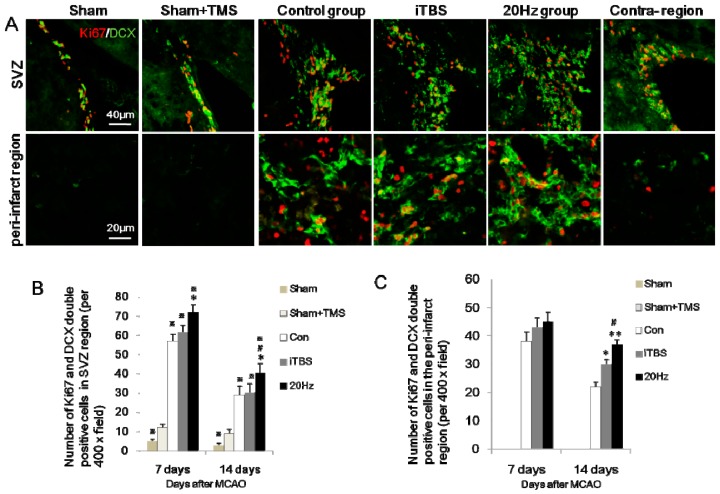
Expression of Ki67/doublecortin (DCX)-positive cells in the ipsilateral subventricular zone (SVZ) and peri-infarct striatum at 14 days after MCAO (**A**) Ki67 colocalized with DCX in cells of the ipsilateral SVZ zone and peri-infarct striatum. After 20 Hz rTMS, numerous cells within the SVZ zone and peri-infarct striatum displayed double-immunopositive signals for Ki67 and DCX, compared with the control group, iTBS group, sham + TMS group, and sham group. Sham and sham + TMS group showed no Ki67/DCX expression in the peri-infarct striatum. The contralateral SVZ showed less Ki67/DCX than the ipsilateral region; (**B**,**C**) Quantification of Ki67/DCX-positive cells in each group. Values are mean ± SD; * *p* < 0.05, ** *p* < 0.001 versus control group; and # *p* < 0.05 versus iTBS group; ^※^ *p* < 0.05 versus sham + TMS group.

To investigate whether high-frequency rTMS promotes neurogenesis, Ki67, a cell cycle marker, and doublecortin (DCX), a marker for migration of neural progenitor cells [18], were used to evaluate migration of NSC in the SVZ after stroke. Numbers of Ki67-immunopositive cells peaked at seven days and decreased thereafter in the ipsilateral SVZ. Treatment with rTMS at 20 Hz, but not iTBS, significantly increased Ki67-positive cells in the ipsilateral SVZ at both seven days (*p* < 0.001) and 14 days (*p* < 0.05) after MCAO. A small number of DCX-positive cells were detected within the SVZ on the non-ischemic side of the experimental and control groups, and within both SVZ of sham-operated animals. In contrast at seven and 14 days after MCAO, DCX-positive cells were evident in larger numbers in the ipsilateral SVZ, spreading to the callosum and striatum of the ischemic hemisphere (Figure 2A). Compared with the control group, iTBS and 20 Hz rTMS significantly increased the number of DCX-positive cells in the ipsilateral SVZ at seven and 14 days after MCAO (all *p* < 0.05). Doubly stained Ki67/DCX-positive cells were sparse in the contralateral SVZ and in sham-operated animals, but became more evident seven and 14 days after MCAO in the ischemic hemisphere (Figure 2A). Sham + TMS group showed more numerous Ki67/DCX in both SVZ than sham group, suggesting that high frequency rTMS enhances neural progenitor cells in sham-operated rats, and 28.15% DCX-positive cells were colocalizated with Ki67. The majority of DCX-positive cells (70.37%) were co-stained with Ki67 in the ipsilateral SVZ region and 42.67% in the peri-infarct striatum, suggesting that ischemia triggers co-expression of Ki67/DCX. Furthermore, Ki67/DCX double-positive cells in the ipsilateral SVZ were significantly more numerous in the 20 Hz group than in either the control group or the iTBS group at both seven and 14 days after MCAO (Figure 2A,B; all *p* < 0.05), suggesting that 20 Hz rTMS enhanced ischemia-induced Ki67/DCX expression. There were no significant differences in Ki67/DCX expression in the contralateral SVZ across these three groups (all *p* > 0.05). Moreover, 20 Hz and iTBS significantly increased Ki67/DCX-positive cells in the peri-infarct striatum at 14 days after MCAO (*p* < 0.05), and 20 Hz showed more numerous Ki67/DCX than iTBS. While sham and sham + TMS group showed no Ki67/DCX expression in the peri-infarct striatum (Figure 2A,C; all *p* < 0.05).

### 2.4. rTMS Increases NSC Proliferation in the SVZ and Peri-Infarct Striatum

**Figure 3:**
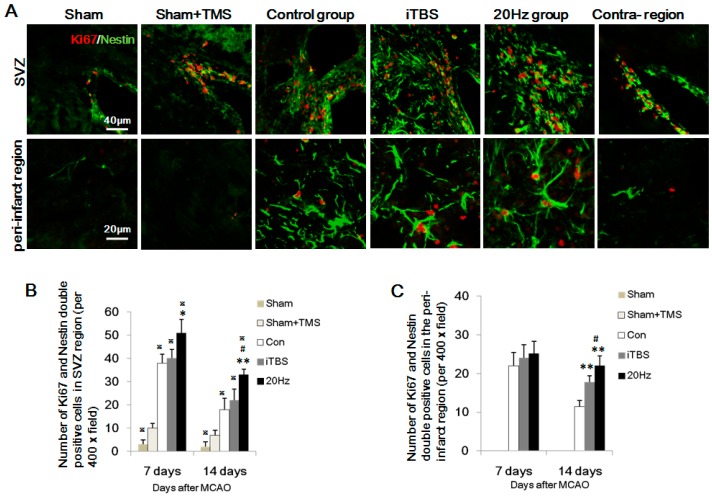
Expression of Ki67/Nestin-positive cells in the SVZ and peri-infarct striatum at 14 days after MCAO. (**A**) Ki67 colocalized with Nestin in cells of the SVZ and peri-infarct striatum. In the 20 Hz rTMS and iTBS groups, there were more Ki67/Nestin-double positive cells in the peri-infarct striatum, compared to the control group. Sham and sham + TMS groups showed no Ki67/Nestin expression in the peri-infarct striatum. The contralateral SVZ showed less Ki67/Nestin-positive cells than the ipsilateral region; (**B**,**C**) Quantification of Ki67/Nestin-positive cells in each group. Values are mean ± SD; * *p* < 0.05, ** *p* < 0.001 versus control group; and # *p* < 0.05 versus iTBS group, ^※^ *p* < 0.05 versus sham + TMS group.

The intermediate filament protein, Nestin, is known as a neural stem/progenitor cell marker and is required for survival and self-renewal [19]. To study whether high-frequency rTMS increases the proliferation of NSC, Nestin was used to label neural stem/progenitor cells in the SVZ and peri-infarct striatum. The number of Ki67 and Nestin-positive cells in the SVZ and peri-infarct striatum reached a peak at seven days and decreased thereafter (Figure 3B,C). Nestin-positive cell numbers were significantly increased in the 20 Hz group compared with the control group at both time points (both *p* < 0.001), and were more numerous than in the iTBS group at 14 days, but not seven days, after MCAO (Figure 3A; *p* < 0.05). The sham + TMS group showed more numerous Ki67/Nestin-positive cells than the sham group. Both of 20 Hz and iTBS increased Ki67/Nestin-positive cells at 14 days after MCAO in the peri-infarct striatum. Moreover, 20 Hz significantly increased Ki67/Nestin-positive cells in the ipsilateral SVZ at seven and 14 days after MCAO (Figure 2A,B, *p* < 0.05), but not iTBS. The sham and sham + TMS groups showed no Ki67/DCX expression in striatum (Figure 2A,C; all *p* < 0.05). Interestingly, 42.15% of the Nestin-positive cells were colocalizated with Ki67 in ipsilateral SVZ and 26.24% of the Nestin-positive cells were also positive for Ki67 in the peri-infarct striatum, indicating that some of the Nestin-positive cells induced by rTMS were newly proliferated cells. These findings suggested that high-frequency rTMS increased the proliferation of NSC in the peri-infarct striatum.

### 2.5. rTMS Promotes NSC Differentiation in the Peri-Infarct Striatum

**Figure 4:**
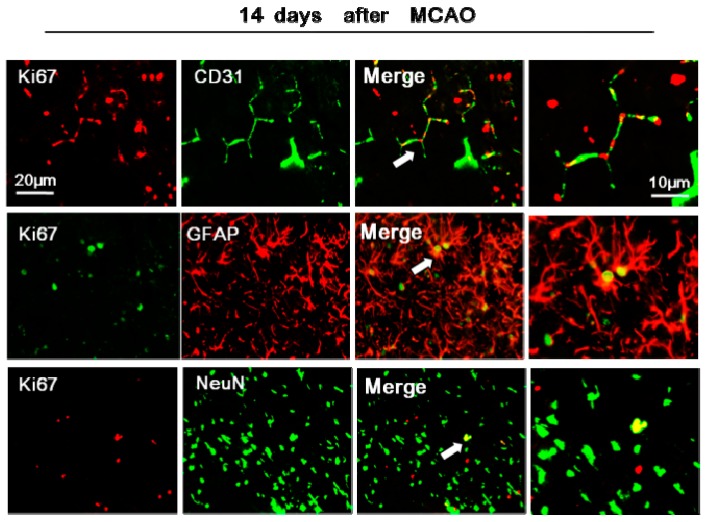
Expression of Ki67/CD31, Ki67/GFAP, and Ki67/NeuN -positive cells in the peri-infarct striatum at 14 days after MCAO. Double-immunofluorescence labeling for Ki67/NeuN, Ki67/GFAP, and Ki67/CD31 (white arrows indicate colocalized cells which are magnified in the right) in the peri-infarct striatum after MCAO. About 6% of NeuN-positive, 18.22% of CD31-positive, and 20.25% of GFAP-positive cells showed co-staining for Ki67 in the peri-infarct striatum of the control group.

To determine whether high-frequency rTMS affects the differentiation of NSC into mature neurons, endothelial cells, or astrocytes, we performed co-staining for Ki67 with NeuN, CD31, or glial fibrillary acidic protein (GFAP) in the peri-infarct striatum at 14 days after MCAO. In the sham-operated and sham + TMS group, less than 3% of NeuN-positive, less than 4% of CD31-positive, and 6% of GFAP-positive cells showed co-staining for Ki67 in the operation side. About 6% of NeuN-positive, 18.22% of CD31-positive, and 20.25% of GFAP-positive cells showed co-staining for Ki67 in the peri-infarct striatum of the control group. The numbers of Ki67/NeuN double positive cells were significantly increased in the 20 Hz group and iTBS group compared with the control group at 14 days after MCAO (*p* < 0.05) and the percent was increased to 8%. However, there were no significant differences between the numbers of cells doubly stained for Ki67/CD31 or Ki67/GFAP across the three groups at any time point (Figure 4, *p* > 0.05).

### 2.6. rTMS Activates BDNF/TrkB Signaling Pathway in Ischemic Rats

**Figure 5:**
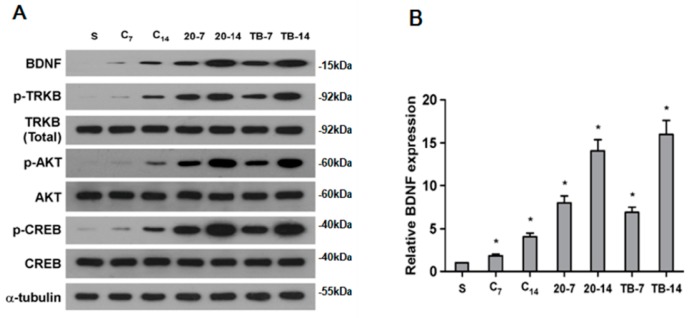
Expression of brain-derived neurotrophic factor (BDNF), phosphorylated-tropomyosin-related kinase B (p-TrkB), TrkB (total), phosphorylated-Akt (p-AKT), Akt, phosphorylated-CREB (cAMP responsive element-binding protein), and CREB in the ipsilateral hemisphere excluding the infarct area. (**A**) Western blot analysis of p-TrkB, TrkB (total), p-Akt, Akt, p-CREB, CREB and BDNF. Protein expression levels were normalized with α-tubulin; (**B**) Quantification of BDNF mRNA expression in the ipsilateral hemisphere excluding the infarct area. BDNF mRNA expression in the 20 Hz rTMS and iTBS groups was significantly higher than that observed in the control group at seven and 14 days after MCAO, while there were no difference between the 20 Hz rTMS and iTBS groups (* *p* < 0.01).

Given that TrkB signaling has been demonstrated to play a key role in NSC migration and proliferation [16,17], we investigated the capacity of rTMS stimulation to activate the TrkB pathway. We found that the expression level of phosphorylated-TrkB increased significantly in the 20 Hz and the iTBS groups at seven and 14 days after MCAO, compared to controls or sham-operated animals (Figure 5A). Consistent with this effect, levels of phosphorylated-AKT and phosphorylated-CREB (cAMP responsive element-binding protein), the downstream targets of phosphorylated-TrkB, were also significantly increased in the 20 Hz group and iTBS group (Figure 5A).

Furthermore, Western blotting and RT-PCR analysis revealed that protein and mRNA levels of BDNF, a key upstream growth factor contributing to TrkB signaling, were both significantly increased in response to rTMS in the 20 Hz and iTBS groups, compared to controls (Figure 5B). Overall, these results further corroborated the role of TrkB signaling in rTMS stimulation-mediated improvements to neurological function and in enhanced neuronal cell proliferation.

## 3. Discussion

In the present study, we demonstrated that high-frequency rTMS improves functional recovery and reduces subacute ischemic brain damage, which is associated with a reduction of infarct volume and an enhancement of neurogenesis. In conjunction with these neuroprotective effects, protein expression levels of BDNF and TrkB activation (phosphorylated-TrkB) were significantly increased by high-frequency rTMS, indicating that this approach is a promising strategy for stroke rehabilitation.

Post-ischemic neurogenesis has been shown to play an essential role in functional recovery [3,20]. Proliferation, migration, and differentiation of NSC have been considered to be amongst the most therapeutically effective strategies following stroke [21]. In this study, we found that 20 Hz rTMS enhanced NSC proliferation in the ipsilateral SVZ, which may be the reason for why high-frequency improves recovery after stroke [22]. This finding corroborates the results of some previous studies, such as that of Guo et al. (2014) [13], who reported that 10 Hz rTMS promotes the Brdu/Nestin double positive cells in the ipsilateral SVZ after focal cerebral ischemia. In addition, we found that both 20 Hz conventional high-frequency rTMS and iTBS significantly increased neural progenitor cell migration and differentiation in the peri-infarct striatum, as evidenced by an increase in the number of cells doubly stained for Ki67/DCX and Ki67/NeuN. However, the stimulation protocols we used did not influence the fate of neural progenitor cells in the contralateral SVZ. The possible reason was that neurogenesis occurs mainly in the peri-infarct region and high-frequency rTMS has an additive effect on it (Figure 1B). Moreover, we observed that Ki67/CD31 double positive cells increased after stroke, but did not increase after high-frequency rTMS in ischemic rats. This phenomenon suggested that stroke activates neurogenesis and angiogenesis, while high frequency rTMS in this study enhanced neurogenesis but could not promote angiogenesis in ischemic rats. Furthermore, we also found the number of Ki67/GFAP positive cells showed no significant differences among 20 Hz, iTBS, and the control group, suggesting that rTMS promoted neural stem cells differentiation to the neuronal lineage more than glial cell lines. Overall, our findings indicated that high-frequency rTMS successfully enhanced neurogenesis in the damaged hemisphere in a rat model of ischemic stroke.

However, alternative mechanisms have also been reported which may account for the beneficial therapeutic effects of high-frequency rTMS. For example, Yoon et al. (2011) [23] demonstrated a therapeutic effect of rTMS on functional recovery in subacute cerebral ischemia associated with an anti-apoptotic mechanism in the peri-ischemic area, rather than via enhanced neural plasticity. These results suggest that in some cases, apoptosis may also play an important role in recovery from ischemia. However, in our data about 8% of Ki67-labeled cells in the peri-infarct striatum were positive for NeuN, a marker of mature neurons that would label newborn cells. This indicated that the increased numbers of newborn neurons were due to the increased number of total surviving cells in the peri-infarct striatum, such as Ki67 and DCX/Nestin/GFAP-double positive cells, which was also a reason for functional recovery by rTMS in ischemic rats [24,25].

Interestingly, neural progenitor cells can synthesize and secrete trophic factors such as BDNF, which has been implicated as a major mediator of endogenous ischemia-induced neurogenesis [26,27,28]. In addition, BDNF accelerates neuroblast recruitment in the SVZ and their migration towards areas of ischemic brain tissue [29]. Recently, high-frequency rTMS at 20 Hz has been reported to stimulate the expression of BDNF in several brain regions in rats, including the hippocampal CA1 and CA3 subfields [14]. It has also been demonstrated that rTMS promotes the proliferation and increases the expression of pERK1/2 and BDNF in hippocampal-derived NSC [30]. Consistent with this, we found that both 20 Hz rTMS and iTBS significantly increased protein expression of BDNF and phosphorylated-TrkB, in parallel with enhanced levels of neurogenesis in the ipsilateral SVZ and peri-infarct striatum. Interestingly, we measured the functional improvement using the MNSS scale which was positively correlated with the degree of BDNF upregulation (*r* = −0.566, *p* < 0.05). Therefore, our results suggest that high-frequency rTMS improves functional recovery by enhancing neurogenesis in the peri-infarct striatum and activating the BDNF/TrkB signaling pathway.

In the present study, 20 Hz rTMS was found to offer a slight improvement in terms of functional recovery and a reduction of brain damage compared to iTBS. The weaker effect of iTBS may be due to its more limited capacity to promote NSC proliferation and migration in the SVZ. We found that iTBS did not increase expression of Ki67 in the ipsilateral SVZ, although it did enhance the expression of DCX, illustrating that iTBS could not enhance newborn neuronal precursor cells in the ipsilateral SVZ and the increasing neuronal precursor cells were inactive. It should be noted that we only tested two rTMS protocols, as this was an exploratory study, so further protocols will need to be investigated in the future. It is of great value to find out optimal rTMS parameters for improving functional recovery after stroke.

## 4. Materials and Methods

### 4.1. Animals

A total of 72 male Wistar rats weighing 200–240 g were used in this study. Rats were housed in the same animal care facility during a 12 h light/dark cycle throughout the protocol with free access to food and water. The experimental work was authorized by the Institutional Animal Ethical Committee of Sun Yat-Sen University and guided by the Guide for the Care and Use of Laboratory Animals from the National Institute of Health (Publication No. 80–23, revised 1996). Wistar rats were suffered for left side transient MCAO surgery under a general anesthesia, as described in a prior study [19,31]. A silicon-coated nylon monofilament was introduced into left middle cerebral artery for 90 min to induce occlusion. Filaments were removed allowing reperfusion of blood to the affected areas. Animals were kept on a heating pad at 37 °C until they completely recovered in order to minimize the pain of each animal as much as possible. After recovery from anesthesia, they were returned to their home cages and had free access to food and water.

### 4.2. Neurobehavioral Evaluation

The neurobehavioral outcome was evaluated using the modified neurological severity score (MNSS) [32,33,34], performed at two, seven, and 14 days after MCAO. There are four tests in the MNSS, including motor, sensory, balance, and reflex tests. Scores from all the tests were summed to give the MNSS a score of 0–18. Mild neurological injury is described as a score of 1–6, moderate injury is described as a score of 7–12, and severe neurological dysfunction as a score of 13–18. Rats were tested by an experimenter who was blind to the group identity of the animal three times, and the average was recorded. Rats with moderate neurological dysfunction (scores of 7–12) at two days after MCAO were selected for use in the subsequent experimental procedures [31].

### 4.3. Repetitive Transcranial Magnetic Stimulation (rTMS)

A magnetic stimulator (CCY-II, Wuhan Yiruide Medical Equipment, Wuhan, China) with a figure-of-eight coil (B9076; 22 mm inner diameter, 90 mm outer diameter, 76 mm combined long axis length, Wuhan Yiruide Medical Equipment, Wuhan, China) was used to stimulate conscious rats at three days after cerebral ischemia. The maximum stimulator output of the magnetic stimulator was 6.0 T. The stimulation site was located over the ipsilateral primary motor cortex (left M1 zone), as determined by a stereotaxic apparatus. For accurate positioning, one side of nonconducting snap fastener (diameter: 6 mm) was sewn on the ipsilateral M1 zone and the other side glued onto the midpoint of the eight-shaped coil, and the rats were fixed in a bag to avoid escaping (Figure 1B). The stimulation area of this coil covered a 1–2 cm^2^ area of the brain [35], including the peri-infarct region. The coil orientation was 45° in relation to the standard lateral orientation, which is the optimal coil orientation for stimulation [36]. Motor evoked potentials (MEPs) were measured at the quadriceps femoris muscle of the right hind limb using electromyography (MedelecSynergy; Oxford Instruments, Surrey, UK), according to a previously reported method [23,37]. The resting motor threshold (RMT) was defined as the lowest stimulator output at which the peak-to-peak amplitude of the MEP was greater than 5% of its maximal amplitude in at least half of the 10 trials.

### 4.4. Experimental Grouping

Ischemic rats were randomly divided into five groups: a 20 Hz group (*n* = 16), an iTBS group (*n* = 16), a control group (*n* = 16), and a sham-operated group (*n* = 12), in which the filament was not inserted into the artery, and a sham + TMS group (*n* = 12), in which sham-operated rats received 20 Hz TMS. Rats in the 20 Hz group received 800 stimuli at 120% RMT (24% of the maximum stimulator output) per day, resulting from 40 trains at 20 Hz for 1 s, with 15 s intervals between trains to prevent overheating of the coil. In the iTBS group, 10 bursts were grouped and repeated every 10 s for a total duration of 191.84 s, resulting in 20 trains with 600 pulses at 80% RMT (16% of the maximum stimulator output) per day [38,39]. Rats in the control group experienced the same experimental manipulations and auditory stimuli by placing an inactive coil in the position normally occupied by the active coil, and orienting the active coil perpendicularly on top of the inactive coil. This resulted in similar acoustic and vibratory sensations without exposing the rats to the magnetic field [40,41]. Animals showed no signs of discomfort. All rats receiving rTMS were stimulated for 10 days during a two-week period, beginning at three days after MCAO with a two-day pause (Figure 1A). The sham-operated group were housed in standard cages supplied with adequate food and water, but received no stimulation.

### 4.5. Tissue Preparation for Histochemistry

For Nissl and immunofluorescence staining, rats were sacrificed after function evaluations at seven and 14 days after transient MCAO (*n* = 5 in 20 Hz, iTBS, and control group; *n* = 3 in sham and sham + TMS group) with an overdose of 10% chloral hydrate and perfused transcardially with 4% paraformaldehyde and 0.9% saline at 4 °C. The brains were removed carefully and fixed in the fixative for 8 h, and then immersed sequentially in 20% and 30% sucrose until they sunk, as we previously reported [31].

### 4.6. Infarct Volume Measurement

Nissl staining was used to measure infarct volume in the ipsilateral hemisphere. Five successive coronal sections at 2.0 mm intervals from Bregma +4.0 to −6.0 mm were selected for quantification of the infarct volume. The volumes of the ipsilateral and the contralateral hemisphere were quantified as previously described [42,43] and the relative infarct volume was expressed as a percentage of the total volume of the contralateral hemisphere. Nissl staining was performed with 0.1% cresyl violet (Sigma-Aldrich, St. Louis, MO, USA) according to the standard procedure. The infarct areas marked by black stars (Figure 1B) exhibited severe and consistent ischemic damage [44]. The areas immediately outside this zone were referred to as the “peri-infarct region” (Figure 1C) [45].

### 4.7. Immunofluorescence Analysis

Double immunofluorescence staining was performed to visualize Ki67, doublecortin (DCX), Nestin, neuronal nuclei (NeuN), CD31, and GFAP. Sections were first pretreated for 5 minutes with hot (85 °C) citrate buffer (0.01 mol/L, pH 6.0) for antigen retrieval, followed by 5% normal goat serum for 1 hour at room temperature. After that, sections were incubated overnight at 4 °C with a mixture of rabbit anti-Ki67 (1:200; Abcam, Cambridge, CA, USA) and guinea pig anti-DCX (1:1000; Millipore, Bedford, MA, USA), or with mouse anti-Nestin (1:200; Millipore), mouse anti-NeuN (1:200; Millipore), mouse anti-CD31 (1:200; Abcam), or mouse anti-GFAP (1:200; Abcam). After rinsing three times in phosphate-buffered saline (PBS) for 5 min each, sections were incubated for 1 h at 37 °C with a secondary antibody: Alexa Fluor 555/488 mouse anti-rabbit IgG (1:1000; Cell Signaling Technology, Danvers, MA, USA), Alexa Fluor 488 goat anti-guinea pig IgG-FITC (1:200; SCBT, Santa Cruz, CA, USA), or Alexa Fluor 488/555 rabbit anti-mouse IgG (1:1000; Cell Signaling Technology). After rinsing, sections were mounted in a fluorescent mounting medium (Fluoroshield with 4′,6-diamidino-2-phenylindole (DAPI), Sigma) Fluorescence signals were examined under a microscope (BX51; Olympus, Japan) and a 488 nm diode laser and a 543 nm diode laser were used for the detection. Negative control sections were incubated with PBS instead of primary antibodies and showed no positive fluorescent signals.

### 4.8. Western Blotting

The rats in each group (*n* = 3) were sacrificed at seven and 14 days after MCAO. Tissues in the ipsilateral hemisphere (excluding the infarct area) were selected for Western blotting. Under deep anesthesia, the brain tissue was rapidly removed and dissected, and protein was homogenized in a cell lysis buffer (Fermentas, Burlington, ON, Canada) containing a complete protease inhibitor cocktail (Thermo, Rockford, AL, USA). Next, 12% sodium dodecyl sulphate-polyacrylamide gels were used to separate equal amounts of protein from each sample which were electrophoretically transferred onto polyvinylidene fluoride membranes (Millipore), The membranes were incubated with rabbit anti-BDNF (1:2000; Abcam, 15 kDa), rabbit anti-TrkB (92 kDa), phosphorylated-TrkB (91 kDa), AKT(56 kDa), phosphorylated-AKT (56 kDa, phospho S473), CREB (37 kDa), phosphorylated-CREB (37 kDa, phospho S133) (1:1000; Abcam), and mouse anti-α-tubulin (1:1000; Cell Signaling Technologies, 50 kDa) overnight at 4 °C after blocking with 5% skimmed milk in Tris-buffered saline with 0.1% Tween 20 (TBST) for 1 h at 25 °C. The membranes were then washed in TBST and incubated for 1 h at room temperature with horseradish peroxidase-conjugated secondary antibody (1:10,000; Abcam). Immunoreactivity was visualized by using the enhanced chemiluminescence method.

### 4.9. Data Analysis and Statistics

#### 4.9.1. Image Analysis and Quantification

All histological images were captured at the same exposure and analyzed using the Image-Pro Plus image analysis software (Media Cybernetics, Silver Spring, MD, USA) by an experimenter who was blind to the group identity of each animal. For cell counting of Ki67/DCX/Nestin/NeuN/CD31/GFAP-immunopositive cells, eight consecutive sections at 240 μm intervals from Bregma 0.20–2.20 mm were analyzed. The regions of interest were defined as a zone with 500 μm width and length in the peri-infarct striatum, which is immediately outside the infarct zone. The numbers of Ki67/DCX/Nestin/NeuN/CD31-immunopositive cells in the peri-infarct striatum (marked by black triangles in Figure 1C) were counted in five non-overlapping fields (425 μm × 320 μm) under 400× magnifications, and were presented as the average cell number on each section. The final cell number calculated for each immunofluorescence marker in each rat was the mean of the average cell number per field across all the sections [46]. For Western blotting, densitometric analysis was performed using the Quantity One software package (Bio-Rad, Hercules, CA, USA).

#### 4.9.2. Statistical Analysis

Numerical data were presented as mean ± SD. Repeated measures one-way ANOVA was used to evaluate the statistical significance of differences in MNSS values after sphericity test assumption (*p* > 0.1). Comparisons of multiple groups were carried out using ANOVA followed by Fisher’s least significant difference test for data regarding relative infarct volumes and the numbers of Ki67/DCX/Nestin/NeuN/CD31/GFAP-positive cells. Pearson bivariate correlation was used to conduct correlation analyses. Statistical analysis was performed using SPSS 16.0 for Windows (SPSS Inc., Chicago, IL, USA). A significance level of *p* < 0.05 was considered statistically significant.

## 5. Conclusions

In conclusion, high-frequency rTMS improves functional recovery possibly by enhancing neurogenesis and activating BDNF/TrkB signaling pathway and conventional 20 Hz rTMS is better than iTBS at enhancing neurogenesis in ischemic rats.

## Abbreviations

rTMS	repetitive transcranial magnetic stimulation
BDNF	brain-derived neurotrophic factor
TrkB	tropomyosin-related kinase B
MCAO	middle cerebral artery occlusion
DCX	doublecortin
GFAP	glial fibrillary acidic protein
NSC	neural stem cell
MNSS	modified neurological severity score
SGZ	subgranular zone
SVZ	subventricular zone
DG	dentate gyrus
iTBS	intermittent theta burst stimulation
LTP	long-term potentiation
LTD	long-term depression
RMT	resting motor threshold
MEP	motor evoked potentials
DAPI	4′,6-diamidino-2-phenylindole
PBS	phosphate-buffered saline
TBST	Tris-buffered saline with Tween 20
CREB	cAMP responsive element-binding protein
ANOVA	analysis of variance
